# Performance evaluation drives organizational change: a case-study from Tuscan Teaching Hospitals

**DOI:** 10.1093/eurpub/ckac131.434

**Published:** 2022-10-25

**Authors:** A Ferrari, M Bonciani, M Vainieri

**Affiliations:** Management and Health Laboratory, Sant'Anna School of Advanced Studies, Pisa, Italy

## Abstract

The 1985 WHO recommendation on caesarean-section (CS) stated that CS rates over 10-15% might be clinically unnecessary. The Performance Evaluation System of Sant'Anna School of Advanced Studies systematically monitors the regional health performance of Tuscany, ITALY. CS rates in Pisa Teaching Hospital (TH) were the highest in Tuscany before 2018 (almost 30%), but then started to decrease, reaching the lowest values in Tuscany, around the WHO threshold (15%). We aimed to assess and demonstrate such performance improvement in Pisa TH, taking the other two THs of Tuscany as comparators. Our outcomes were NTSV CS rates (nulliparous, term pregnancy, singleton, vertex), total CS rates, Robson Class 1 CS rates, NTSV episiotomy rates, and operative delivery rates, computed annually from 2016 to 2020 in the three THs of Tuscany. We obtained data from regional administrative databases. We performed difference-in-differences to compare the average change from the pre- (2016-2017) to the post-period (2018-2020) in the outcomes between Pisa TH and the other two THs, assuming that Pisa TH implemented organizational changes at the end of 2017 to improve the hospital performance on CS. Comparing Pisa TH with the other two THs, we found a significant pre-to-post reduction in NTSV CS rates (p < 0.001), total CS rates (p = 0.016), Robson Class 1 CS rates (p = 0.039), and episiotomy rates (p = 0.059), while no significant change emerged for operative deliveries. As a result, after 2017, Pisa TH reached significantly lower NTSV CS rates (p < 0.001) and Robson Class 1 CS rates (p = 0.027) and non-significantly higher total CS rates and episiotomy rates compared to the other two THs. This case study proved beyond a mere descriptive assessment that a statistically significant performance improvement in CS and episiotomy rates occurred in Pisa TH after 2017 following organizational changes. Comparison with the pre-period trend and the other two THs of Tuscany made these findings robust and reliable.

**Key messages:**

• The Performance Evaluation System allowed to benchmark the performance of Tuscan THs, revealing the worst practice of Pisa TH on CS, and fostering organizational changes to be explored qualitatively.

• Such organizational changes improved hospital performance on CS rates and on the maternity pathway in general, eventually making Pisa TH the best performer on CS in Tuscany.

